# Age-related creatinophosphokinase and myocardial fibrosis changes in patients with muscular dystrophy

**DOI:** 10.1186/1532-429X-17-S1-P293

**Published:** 2015-02-03

**Authors:** Antonildes N Assunção, Carlos H Rassi, Ricardo Oguro, Rodrigo D Melo, Jacob Sessim Filho, Jose B Araujo Filho, Marly C Silva, Zilda M Meira, Roberto Kalil, Carlos E Rochitte

**Affiliations:** 1 Heart Institute, InCor, University of Sao Paulo Medical School, Sao Paulo, Brazil; 2 Federal University of Minas Gerais, Belo Horizonte, Brazil; 3 Axial Centro de Imagem, Belo Horizonte, Brazil

## Background

Duchenne (DMD) and Becker (BMD) muscular dystrophy (MD) are characterized by progressive peripheral muscular damage. The cardiac involvement is high and often complicated by severe heart failure and high mortality. Cardiovascular magnetic resonance (CMR) can identify early stages of cardiomyopathy, as presence of myocardial fibrosis (MF). Several studies have shown negative correlation between creatinophosphokinase (CPK) levels and worst stages of peripheral muscular dystrophy, but have not evaluated their possible association with myocardial damage. Our objective was to investigate CPK levels and magnitude of myocardial damage in patients with MD.

## Methods

We analyzed 70 pts DMD and 6 BMD with confirmed MD. All patients underwent cine-MR with SSFP sequence for left ventricle (LV) ejection function (EF) evaluation and late gadolinium enhancement (LGE) for MF detection. LV volumes and function were measured by Simpson's method on the short-axis images. MF was measured as the area with signal intensity above 5 standard deviation of mean normal myocardium and expressed as percent of LV mass. All analyses were performed using CVi42 software (Circle CVi, Calgary, CA). Comparison between groups was made using the unpaired *t* test and Mann-Whitney test for continuous variables with normal and non-normal distribution, respectively. To multivariate analysis of variables measured at different scales was performed a standardized variable, each rescaled to have a mean of zero and a standard deviation of one. Correlation analysis was performed using the Spearman correlation coefficient. All statistical analyses were performed using Stata version 12 (StataCorp). A 2-tailed P value < 0.05 was considered significant.

## Results

**Figure 1:**
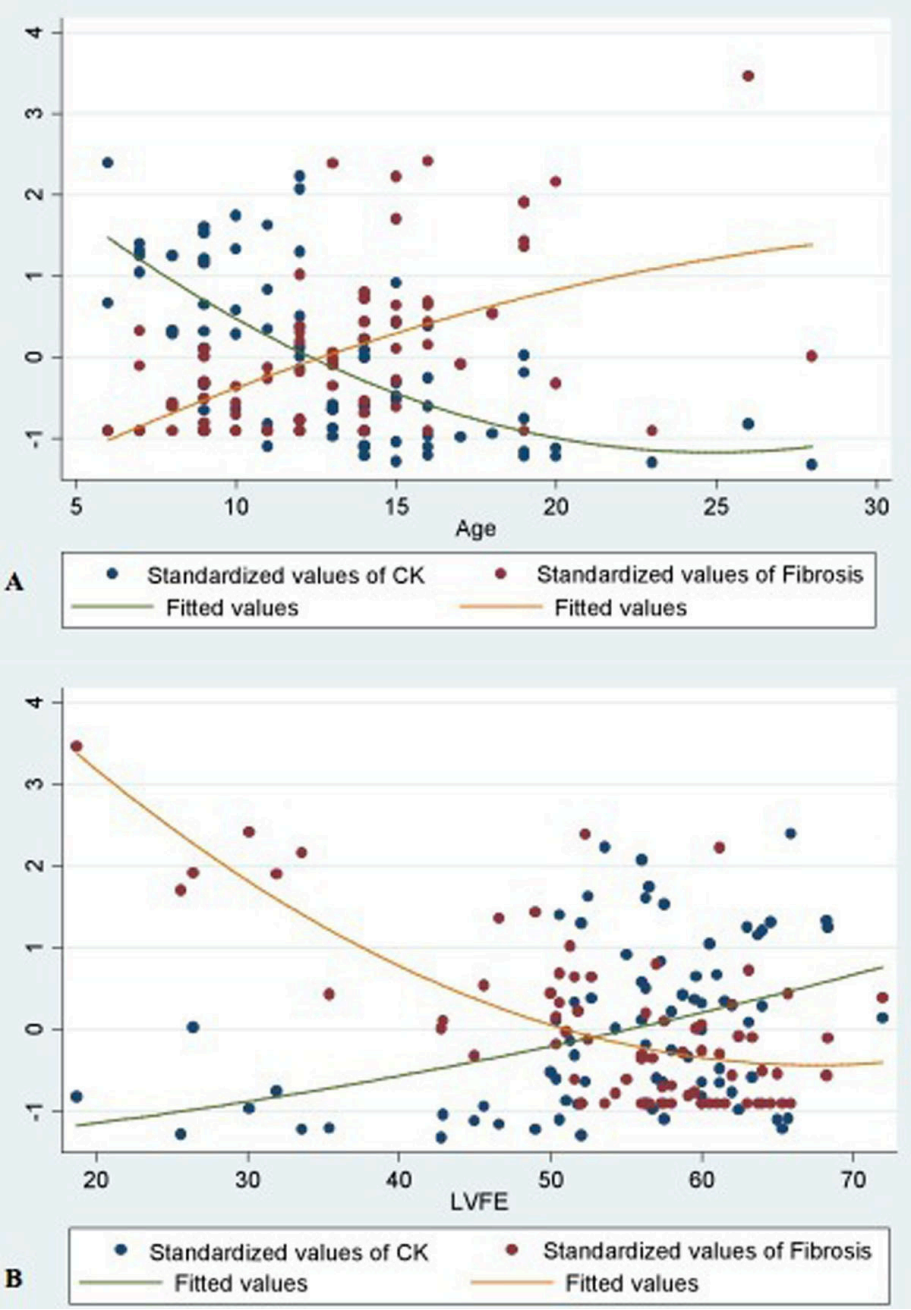
Standardized data of CK and fibrosis by CMR for Age (A) and LVFE (B).

Baseline characteristics are shown on Table 1. Standardized data for age and for LVEF by CMR are shown in Figure 1. Thirteen patients (17.1%) presented LVEF dysfunction (LVD) and, in comparison with patients with normal function, were significantly older (19.2 vs. 11.8 years, p<0.001), with higher CK levels (11552.7 vs 2682.3, p<0.001), lower LVEF (36.4% vs. 58.3%, p<0.001) and MF as percent of LV mass (30.0% vs 8.6%, p<0.001). LVEF showed moderate correlation with CK levels (r=0.479, p<0.001), moderate and inverse correlation with age (r=-0.590, p<0.001), and good and inverse correlation with MF (r=-0.669, p<0.001). Additionally, after using multivariable logistic regression, age, CK levels and MF continued to be independent variables for predicting LVD (LVEF < 50%).

**Table 1:** Baseline characteristics of patients with muscular dystrophy.

	All (n=76)	Normal LVEF (n=13)	LVD (n=13)	p
Age, years ± SD	13.0 ± 4.4	11.8 ± 3.3	19.2 ± 3.9	<0.001

BMI, kg/m2 ± SD	19.6 ± 4.4	18.9 ± 4.1	22.9 ± 4.1	0.002

CK, ± SD	10035.4 ± 7305.6	11552.7 ± 7040.9	2682.4 ± 2603.4	<0.001

LVEDVI, ml ± SD	70.7 ± 32.7	62.5 ± 17.5	110.5 ± 55.1	<0.001

LVESVI, ml ± SD	33.8 ± 28.2	25.6 ± 7.9	73.7 ± 50.5	<0.001

LVEF, % ± SD	54.5 ± 10.2	58.3 ± 5.2	36.4 ± 9,5	<0.001

LV mass, g ± SD	80.8 ± 40.3	70.8 ± 29.6	128.9 ± 50.9	<0.001

Fibrosis, % ± SD	12.3 ± 13.5	8.6 ± 9.8	30.0 ± 14.9	<0.001

## Conclusions

In this group of MD patients, we have demonstrated an inverse correlation between CK levels and extent of myocardial fibrosis standardized by age and left ventricular ejection fraction. Moreover, independent predictors of left ventricular dysfunction were found to be: age, CK levels and myocardial fibrosis extent.

## Funding

N/A.

